# TiO_2_ Nanomaterials Non-Controlled Contamination Could Be Hazardous for Normal Cells Located in the Field of Radiotherapy

**DOI:** 10.3390/ijms21030940

**Published:** 2020-01-31

**Authors:** Yidan Wang, Allan Sauvat, Celine Lacrouts, Jérôme Lebeau, Romain Grall, Marie Hullo, Fabrice Nesslany, Sylvie Chevillard

**Affiliations:** 1 Commissariat aux Energies Atomique et Alternative, Fundamental Research Division, Jacob Institut, Laboratoire de Cancérologie expérimentale, CEA, F-92265 Fontenay-aux-Roses, France; yidan86@gmail.com (Y.W.); celine.lacrouts@cea.fr (C.L.); Jerome.lebeau@cea.fr (J.L.); romain.grall@cea.fr (R.G.); marie.hullo@cea.fr (M.H.); 2 Gustave Roussy, Institut National de la Santé et de la Recherche Médicale, U1138, 94800 Villejuif, France; allan.sauvat@gustaveroussy.fr; 3 Laboratoire de Toxicologie Génétique, Institut Pasteur de Lille, Université de Lille, EA 4483, 59000 Lille, France

**Keywords:** TiO_2_ nanoparticles, photocatalysis, radiation sensitivity

## Abstract

Among nanomaterials (NMs), titanium dioxide (TiO_2_) is one of the most manufactured NMs and can be found in many consumers’ products such as skin care products, textiles and food (as E171 additive). Moreover, due to its most attractive property, a photoactivation upon non-ionizing UVA radiation, TiO_2_ NMs is widely used as a decontaminating agent. Uncontrolled contaminations by TiO_2_ NMs during their production (professional exposure) or by using products (consumer exposure) are rather frequent. So far, TiO_2_ NMs cytotoxicity is still a matter of controversy depending on biological models, types of TiO_2_ NMs, suspension preparation and biological endpoints. TiO_2_ NMs photoactivation has been widely described for UV light radiation exposure, it could lead to reactive oxygen species production, known to be both cyto- and genotoxic on human cells. After higher photon energy exposition, such as X-rays used for radiotherapy and for medical imaging, TiO_2_ NMs photoactivation still occurs. Importantly, the question of its hazard in the case of body contamination of persons receiving radiotherapy was never addressed, knowing that healthy tissues surrounding the tumor are indeed exposed. The present work focuses on the analysis of human normal bronchiolar cell response after co-exposition TiO_2_ NMs (with different coatings) and ionizing radiation. Our results show a clear synergistic effect, in terms of cell viability, cell death and oxidative stress, between TiO_2_ NMS and radiation.

## 1. Introduction

The use of nanomaterials (NMs) has drastically increased in the last decades and they are now present in a huge number of engineered products. Among NMs, titanium dioxide (TiO_2_) is one of the earliest and most manufactured NMs worldwide [1] because of its brightness and high refractive index, with an annual world production of 10,000 t [2,3]. The bulk form of TiO_2_ is biologically inert, however at the nanosized scale it presents specific physical, chemical and biological properties, in part related to its surface reactivity [4]. These characteristics make it attractive in many fields of the industry: papers, inks, medicines, food products and toothpastes, but the main fields using TiO_2_ NMs are skin care products, cosmetics, textiles and plastics as an ultraviolet radiation scavenger.

Regarding human risk, exposures may occur during both manufacturing and using products, via any of the major point-of-entry routes. Indeed, if inhalation and dermal routes are the most important ways of NMs exposure/contamination, the oral one cannot be neglected so far. It is estimated that more than 150 TiO_2_ NMs-containing cosmetic items still can be detected a long time after skin application, and that a typical diet leads to the ingestion of 300–400 mg/day of TiO_2_. However, contamination by TiO_2_ inhalation has gained special concern [5] since, depending on the NMs lifespan and on how the NMs are attached/included into their matrix, NMs could be released in ambient atmosphere. After inhalation, NMs are distributed differentially along the respiratory tract according to their size. With a diameter of 20 nm, they preferentially deposit in the alveolar regions of the lungs; below that size, increasing fractions deposit in the airways of the head and thorax [6]. After their initial adsorption, TiO_2_ NMs can translocate and thus can distribute within the entire body through systemic distribution. However, data concerning TiO_2_ NMs absorption by humans through the respiratory tract are not available yet.

TiO_2_ NMs have specific physical and chemical properties, and their ability to get photoactivated, which is one of them, is very attractive for many applications related to UV filtering, air and water remediation. Thus, TiO_2_ NMs are included in numerous products that should be microbial free. Their effectiveness in decontaminating and killing microorganisms is related to their capacity to generate reactive oxygen species (ROS). These mechanisms are well-documented [7,8]. Briefly, when the energy of incident photons is larger than the band gap of NMs (3.2 eV for TiO_2_, i.e., UV light), electrons can be provided to the conduction band that leads to the presence of holes in the valence band. Reactions between electrons or holes and surrounding molecules lead to ROS production. Li et al. investigated the mechanisms related to ROS production for TiO_2_ NMs compared to other metal-oxide NMs [9] and three types of ROS were detected: superoxide radicals, hydroxyl radicals and singlet oxygen. 

Regarding medical applications, and especially cancer treatments, the photocatalytic activation of TiO_2_ NMs generates ROS that are cyto- and genotoxic, leading to tumor cells death through induction of membrane alteration, lipid bilayer disruption and oxidized DNA damages. The process of ROS production through the photoactivation of TiO_2_ NMs was shown to be effective after exposure to non-ionizing radiation, either visible light or UVA [10,11]. Taking advantage of this ROS generation, promising outcomes are expected for TiO_2_ NMs-UV photodynamic therapy for endobronchial and esophageal tumors [12]. In comparison, only a few studies have addressed the photocatalytic activity of TiO_2_ after exposure to ionizing radiation (IR) at higher energies, such as X-rays or γ-rays. Even if the effect is less important compared to UV illumination, due to the weaker absorption of photons at higher energies, a photo-electrochemical process still occurs under X-ray exposure [13]. In the medical field, the exposure of titanium oxide-based nanotubes or earth doped titania nanoparticles increases the efficiency of X-ray irradiation to kill tumor cells *in vitro* [14,15].

Regarding hazards and risks for human health, cyto- and genotoxicity of TiO_2_ NMs are still a matter of controversy. Depending on the studied biological models (many different *in vitro* cell types), the type of TiO_2_ NMs (especially its crystalline structure (rutile and anatase), size and shape), the techniques used according to the biological endpoints, positive and negative results have been published [6,16,17]. However, in 2006, the International Agency for Research on Cancer classified titanium dioxide TiO_2_ in the group of substances “possible carcinogens in humans” (group 2B) if inhaled whatever the form (IARC 2006), mainly due to two studies showing that at very high concentrations it can induced respiratory tract cancer in exposed rats [18,19]. Moreover, due to their photoactivation that generates cytotoxic ROS, TiO_2_ NMs could indeed induce deleterious effects under radiation exposure. However, to prevent the deleterious effect induced by ROS production ultraviolet (UV) radiations, UV radiation scavenger could be used and cosmetics industries have started using new-generations of TiO_2_, coated with a thin layer of silica or alumina [20]. However, no study has been conducted to show the potential interest of using such new compounds for protecting healthy tissues exposed to high doses of ionizing radiation during the time course of radiotherapy.

Since inhalation is an important route of contamination and knowing that radiotherapy is an important modality for head and neck cancer treatment, in the present work we addressed the question whether pulmonary normal tissue contaminated with TiO_2_ NMs could have a different sensitivity to IR. We exposed the human bronchial epithelial cell line (16HBE14o-) to different TiO_2_ NMs and studied if it conferred modifications in radiation sensitivity. The dose of 4 Gy for γ-rays was chosen as being compatible with daily doses, which are used in conventional radiotherapy and as being a low cytotoxic in the chosen cellular model. Regarding the concentrations of TiO_2_ NMs, they are fully compatible to amounts expected in case of lung contamination after inhalation [21].

Overall, we observed a synergistic cytotoxic effect between non-coated and coated TiO_2_ NMS and radiation exposure that is characterized by an increase in cell mortality and in oxidative stress together with a decrease in cell proliferation. Contaminations by NMs presenting a photocatalytic activity, such as TiO_2_ NMs, which are widespread in usual consumer products, could be involved in abnormal healthy tissue radiation sensitivity sometimes observed during the time course of radiotherapy.

## 2. Results

### 2.1. TiO_2_ NMs Internalization into Cells

**Figure 1:**
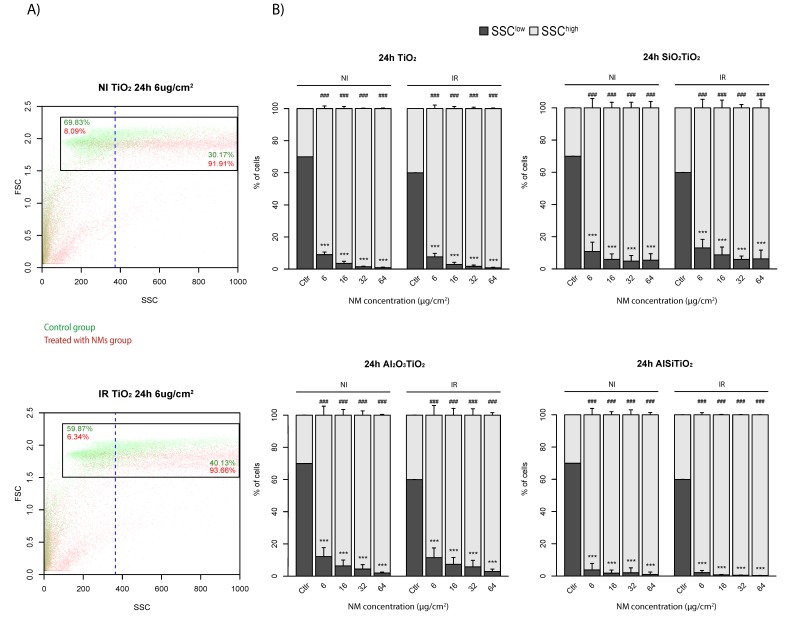
Internalization of nanomaterials (NMs) into 16HBE14o- cells. Cells are platted in 24 wells plates at 4.5 × 10^4^ cells per well, and maintained in control conditions (Ctlr), or in the presence of 6, 16, 32 or 64 μg/cm^2^ of the different type of TiO_2_. (**A**) Flow cytometry are performed 24 h later, and internalization is measured by the increase of SSC for TiO_2_, SiO_2_TiO_2_, Al_2_O_3_TiO_2_ and AlSiTiO_2_. A threshold is defined at 70% for non-irradiated cells, and 60% for irradiated cells regarding the pattern of FSC/SSC for the control groups, and (**B**) the results are reported for each type of NMs. (means ± S.E.M., *n* = 3, *** p < 0.001 (Welch *t*-test), as compared to the percentage of SSC^low^ of the control group, *### p* < 0.001 (Welch *t*-test), as compared to the percentage of SSC^high^ of the control group).

The first step before cytotoxicity measurement following exposure to nanoparticles is to attest that they actually enter into the cells. Nanoparticles entrance into cells is associated with a modification of intracellular granularity and can be measured by the deviation of the SSC channel [22]. In this aim, and according to the pattern of the non-irradiated and the irradiated control groups, a threshold was defined to have 70% and 60% of SSC^low^ cells, respectively (Figure 1A). The same thresholds were then applied to the samples treated with the different concentrations of the four types of TiO_2_ NMs, according if they are irradiated or not. A significant increase in the percentage of SSC^high^ cells was observed 24 h after treatment with all types of TiO_2_ NMs, representing an increase in cell granularity (Figure 1B), even for the lowest concentration (Figure 1A,B), indicating their internalization into the cells. Moreover, this internalization persisted in time, since it still could be detected 48 h and 72 h after treatment, no matter the type of TiO_2_ NMs (Figure S1a,b).

### 2.2. TiO_2_ NMs and Radiation Induced Cell Death

Once the entrance of the NMs into cells was confirmed, cell death of the 16HBE14o-cell line was then measured, by the uptake of 3,3′-Dihexyloxacarbocyanine Iodide (DiOC) and propidium iodide (PI). Indeed, DiOC is a selective fluorescent membrane potential-sensitive dye that accumulates in the intact mitochondria of living cells, and PI is a fluorescent dye that is uptaken only by dead cells. Using these two dyes concomitantly permits then to separate precisely the different steps of dying cells: indeed, DiOC^+^ /PI^−^ represent living cells since they still present a membrane potential detectable by DiOC and exclude PI, the double negative cells represent the apoptotic cells, which have already lost their membrane potential but are not permeable yet to PI, and the PI^+^ cells represent dead cells [23]. 

**Figure 2:**
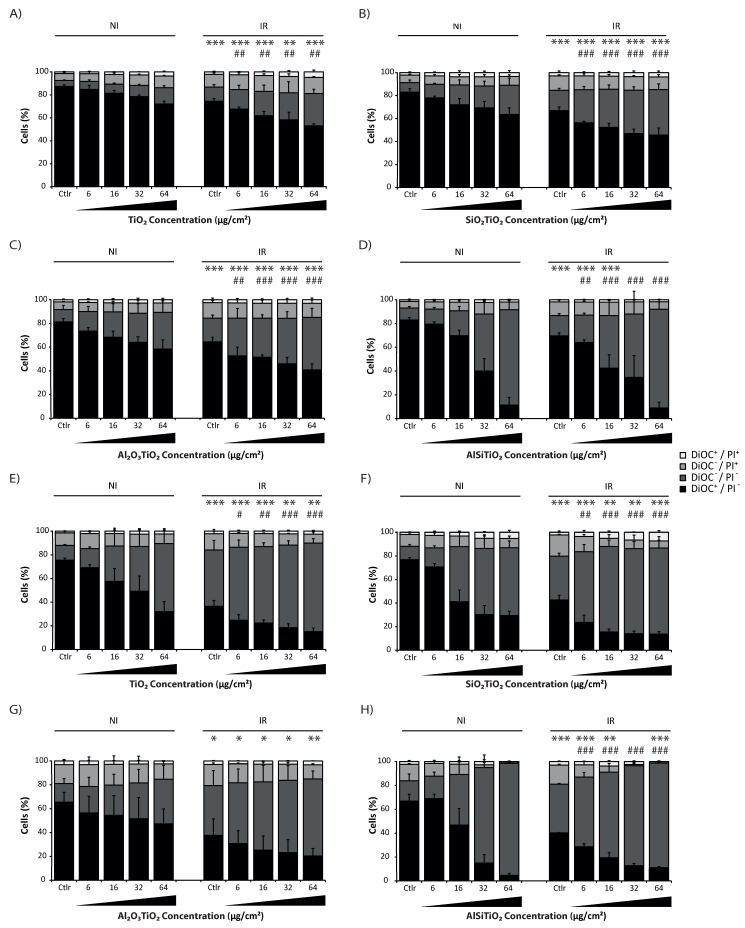
Cell death assay performed 72 h and 7 days after TiO_2_ treatment combined or not with 4 Gy irradiation. Human bronchial epithelial 16HBE14o- cells are maintained in control conditions (Ctlr) or treated with 6, 16, 32 or 64 μg/cm^2^ of (**A**,**E**) titanium dioxide (TiO_2_), (**B**,**F**) Silica coated titanium dioxide (SiO_2_TiO_2_), (**C**,**G**) Aluminum coated titanium dioxide (Al_2_O_3_TiO_2_) or (**D**,**H**) double silica and aluminum coated titanium dioxide (AlSiTiO2), and irradiated or not. Cells are maintained in culture for 72 h (**A**–**D**) or 7 days (**E**–**H**) after treatment and then co-stained for the cytofluorometric detection of cell death with DiOC6(3) and propidium iodide. Representative dot plots and quantitative data are reported (means ± S.D. *n* = 3, except for A) *n* = 2). * *p* < 0.05, ** *p* < 0.01, *** *p* < 0.001 (Student’s *t*-test), as compared with cells maintained in same conditions but non-irradiated. ^#^ *p* < 0.05, ^##^ *p* < 0.01 and ^###^ *p* < 0.001 (Student’s *t*-test), as compared with IR cells maintained in Ctlr condition.

Thereby, cell death assays are performed 24, 48, 72 h and 7 days after treatment with the NMs or/and 4 Gy irradiation (Figure 2 and Figure S2). Overall, independent of the type of treatment (NMs ± irradiation), a dose-dependent cell death was already detectable at 24 h, but increased in time (Figure S2). Seventy-two hours after treatment, 16HBE14o- cells still show a good resistance to 4 Gy irradiation alone (25.5–35% of dead cells; Figure 2). Surprisingly, non-coated TiO_2_ NMs alone already induced a dose-dependent cell death, even reaching the same toxicity than the irradiation alone at the highest concentration tested (64 µg/cm^2^) after 72 h of exposure (Figure 2A). However, the most significant deleterious effect for the non-coated TiO_2_ NMs was observed when cells were co-exposed to 4 Gy irradiation. Indeed, cell death was significantly increased in a dose-dependent manner (until 47% of cell death at 64 µg/cm^2^ + IR) compared to the only-irradiated control group (25.5% of cell death) or to the cells only treated with the non-coated TiO_2_ NMs (27.95% at 64 µg/cm^2^). This mortality still increased in time since 7 days after treatment, even for the irradiated control group without any NMs, it reached 63.4% of cell death (Figure 2E). However, when cells were treated with non-coated TiO_2_ NMs combined with 4 Gy irradiation, a significant augmentation, which is always dose-dependent, was observed (until 84.87% of cell death at 64 µg/cm^2^ combined with 4 Gy irradiation). 

Overall, due to their rapid internalization and accumulation into cells, TiO_2_ NMs induce cytotoxicity, without any photoactivation, due to their intrinsic physical and chemical properties. However, under 4 Gy irradiation, the photocatalytic properties of the TiO_2_ NMs were activated and potentiated the toxicity of the TiO_2_ NMs, which accentuated cell death in time.

### 2.3. Coated TiO_2_ NMs and Radiation Induced Cell Death

In order to avoid this photocatalytic effect, TiO_2_ NMs used were now coated with a different kind of layers, which are supposed to reduce their photocatalytic activity and thus their reactivity upon radiation [20]. Therefore, three different coatings were tested to study their ability to protect cells from the photocatalytic damages induced by TiO_2_ NMs upon 4 Gy irradiation: TiO_2_ NMs coated with silicon, aluminum or both of them.

In the cases of the silicon (SiO_2_TiO_2_) and aluminum (Al_2_O_3_TiO_2_) coatings, cell death assays measured 24, 48, 72 h and 7 days after treatment by flow cytometry also show a dose-dependent cell death detectable from 24 h after treatment (Figure 2 and Figure S2). However, unexpectedly, 72 h after treatment, these two coated TiO_2_ NMs induced a higher level of mortality than the non-coated TiO_2_ NMs at the same concentration under the non-irradiated condition (at 64 µg/cm^2^, SiO_2_TiO_2_ and Al_2_O_3_TiO_2_ induced 36.5% and 41.55% of cell death respectively, compared to 27.95% for the non-coated TiO_2_ NMs; Figure 2A–C). However, upon 4 Gy irradiation, the differences between the non-coated TiO_2_ NMs and the coated ones became less notable, even if the coated ones still induced a higher toxicity than the non-coated TiO_2_ NMs (at 64 µg/cm^2^, 54.3% and 59.2% of cell death SiO_2_TiO_2_ and Al_2_O_3_TiO_2_ respectively, compared to 46.9% for the non-coated TiO_2_ NMs).

Seven days after treatment without irradiation, the cell death level induced by the coated and non-coated NMs became roughly the same (at 64 µg/cm^2^, 70.57% of cell death for SiO_2_TiO_2_, compared to 68% for non-coated TiO_2_ NMs), and Al_2_O_3_TiO_2_ became even less toxic than the non-coated TiO_2_ NMs (52.65% of cell death; Figure 2E–G). After irradiation, there were no longer differences between the coated NMs and the non-coated one (at 64 µg/cm^2^, 86.27% of cell death for SiO_2_TiO_2_ 88.93% for Al_2_O_3_TiO_2_ and 84.87% for non-coated TiO_2_).

These results show that without any irradiation, the coated NMs induced a higher physical toxicity in a shorter time than the non-coated TiO_2_, even if in the long term they finish to reach the same level of mortality, except for Al_2_O_3_TiO_2_, which seemed less toxic. In all cases, under an irradiation exposure, these two tested-coatings provide no protection against the induction of TiO_2_ NMs’ photoactivity by highly energetic ionizing radiation.

The last tested coating was the double component silicon and aluminum made TiO_2_ NMs (AlSiTiO_2_). Only 24 h after treatment, a drastic dose-dependent increase of cell death occurred, even without any irradiation (Figure S2D). Seventy-two hours after treatment, the concentration of 32 µg/cm^2^ reached the same level of cell death than the one induced by 64 µg/cm^2^ + IR with the other types of TiO_2_ NMs. The NMs induced such a high level of toxicity that the addition of 4 Gy irradiation had no further effect beyond 32 µg/cm^2^ (at 32 µg/cm^2^, 60% of cell death for the non-irradiated AlSiTiO_2_, and 65.22% for the irradiated ones; Figure 2D). Furthermore, AlSiTiO_2_ already reached its maximal toxicity at 24 h, since there was no difference among 24, 48, 72 h and 7 days after treatment (Figure 2D,H and Figure S2). 

Altogether, these results show that the three different tested-coatings, developed to minimize the side effects of non-ionizing UVs on TiO_2_ NMs, were inefficient against deleterious effects induced by the photocatalytic activation of these NMs under ionizing radiation sources such as radiotherapy. Moreover, the coating itself induced toxicity, by their easy internalization by cells and probably due to its own chemical properties. It is important to notice that the NMs stayed accumulated into cells, at least until 7 days after their introduction into cells, and that cell death induced by the NMs themselves accentuated in time. The double coating, which is supposed to be less toxic or at least more protective against the irradiation, shows a very high toxicity into cells, even at low concentrations. It can be supposed then that the more the NMs include chemical components, the more they will interfere with the normal behavior of the cell and be toxic to it.

### 2.4. Cell Growth

**Figure 3:**
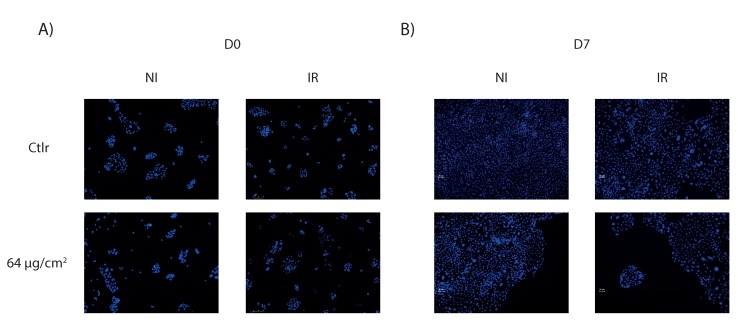

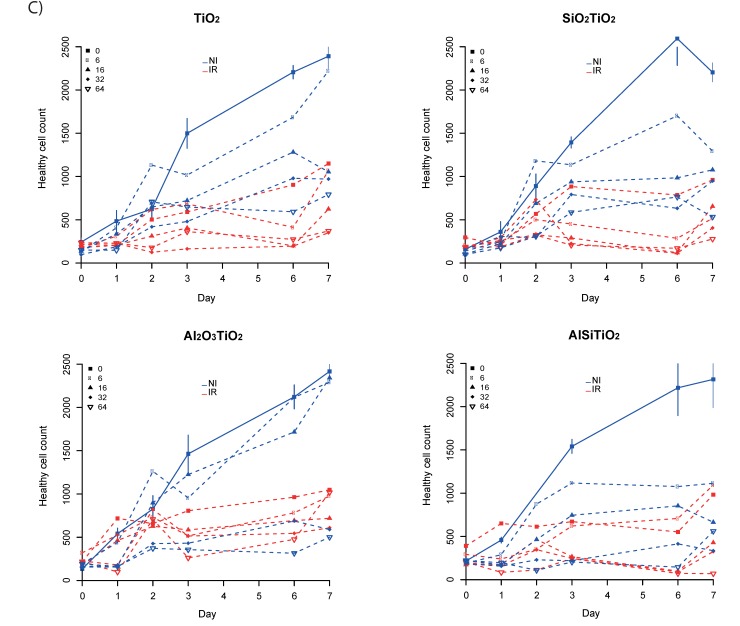
Cell growth after treatment by TiO_2_ NMs followed by microscopy. 16HBE14o- cells are platted in six wells plates at 2.5 × 10^4^ and treated 24 h after with no NMs, 6, 16, 32 or 64 μg/cm^2^, combined or not with 4 Gy irradiation. Microscopy pictures are taken immediately (10× magnification) (**A**) at day 0 until (**B**) day 7 after treatment on fixed cells, stained with Hoechst 33342. (**C**) The average of the cell number is obtained for each type of TiO_2_ until day 7 (the standard deviation is only shown for the non-irradiated control group for figure readability).

Cell death was also assessed by microscopy, following the cell growth during 7 days after treatment with the different TiO_2_ NMs combined or not with 4 Gy irradiation (Figure 3A,B). Cells were platted at 2.5 × 10^4^ cells per well and treated the day after (D0). Cell number was determined by automated image analysis from five pictures per condition, means of these five measurements were reported. For more clarity, only the standard deviation of the non-irradiated control group was shown (Figure 3C). After one week, a dose-dependent cell growth inhibition was observed. As expected, the non-coated TiO_2_ NMs, the SiO_2_TiO_2_ and Al_2_O_3_TiO_2_ interfered with cell growth equivalently, as cells treated with these NMs presented roughly the same pattern of growing. Under 4 Gy irradiation, there was no protection from the silicon or the aluminum coating compared to the non-coated TiO_2_ NMs: a significant inhibition of the cell number could be observed compared to the non-irradiated cells, in a dose-dependent manner. These results also confirmed that AlSiTiO_2_ NMs appeared to be the most toxic for 16HBE14o- cells, since even with the lowest concentration and in absence of irradiation, the cell number was already halved. At 64 µg/cm^2^ with irradiation, AlSiTiO_2_ NMs inhibited almost the total cell growth. It should be noticed that the small differences observed between microscopy and the cell death assays could be explained by the fact that in cytometry, results reflect the percentage of alive and dead cells on a defined number of cells, whereas in microscopy, the totality of living cells is reported here.

These results confirmed the previous observations, knowing that TiO_2_ NMs (coated or not) prevent the normal division of cells, by inducing a toxicity that is dose-dependent. The different coatings tested procured no protection against the photoactivation of TiO_2_ NMs under an irradiation exposure, which had a long term impact on cells.

### 2.5. Qualification of the TiO_2_ NMs Effect on the Radiation Response

**Figure 4:**
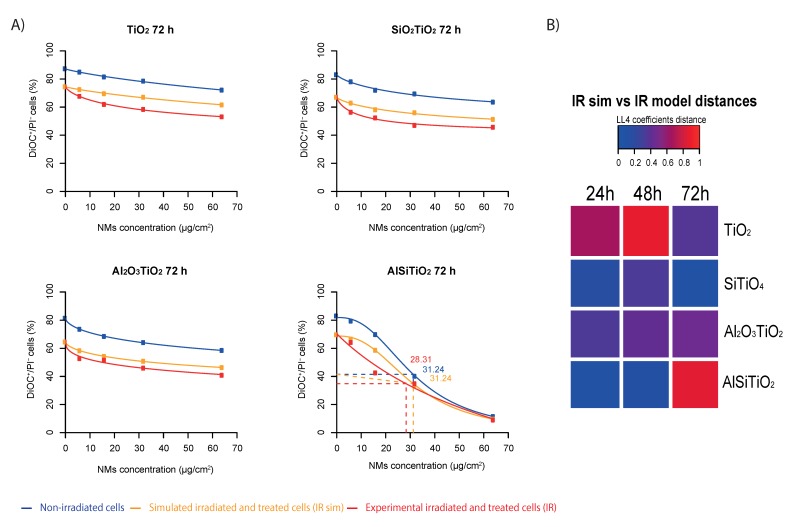
Synergy study between each type of TiO_2_ and 4 Gy irradiation. (**A**) Log-logistic model were built (i) to simulate the theoretical effect between each type of NMs and 4 Gy irradiation, and (ii) to fit the real effect obtained experimentally. The dotted lines: in the case of AlSiTiO2, the synergistic effect was detectable until around 30 µg/cm2. (**B**) Heat map representing a higher or lower effect of experimental toxicity of NMs combined with 4 Gy irradiation versus the theoretical toxicity for 24, 48 and 72 h after treatment.

In order to investigate if there is a synergistic effect between the NMs and the irradiation, a mathematical model [24] simulating the theoretical cytotoxicity of 4 Gy irradiation added to the different types of TiO_2_ NM was performed. This model, based on the results of cell death obtained with the different conditions separately, provided a theoretical mortality induced by “NMs alone” + “4 Gy IR alone”, and compared it to the real one observed experimentally (Figure 4A and Figure S3). Thereby, 72 h after treatment with NMs only, the same tendency of decrease in the percentage of alive cells was observed for the non-coated TiO_2_ NMs, SiO_2_TiO_2_ and Al_2_O_3_TiO_2_, as already mentioned before. However, when cells were exposed to 4 Gy irradiation after treatment, the observed percentage of cells that still remain alive was lower for all the concentrations tested compared to the ones obtained with the theoretical “NMs alone” + “4 Gy IR alone” model. These results highlight that non-coated TiO_2_, SiO_2_TiO_2_ and Al_2_O_3_TiO_2_ NMs do not have a behavior of “simple” nanoparticles, but act in a synergistic way with an irradiation exposure, and induce a cell death higher than what can be expected.

In the case of AlSiTiO_2_, the synergistic effect was detectable until around 30 µg/cm^2^ (dotted lines), but beyond this concentration, which kills 50% of the cells, the NMs induced such toxicity by themselves that there was no difference anymore between the non-irradiated cells, the simulated cell death and the experimental results.

It should be noticed that the non-coated TiO_2_ NMs already showed a synergistic effect with the 4 Gy IR from 24 h after irradiation, with a maximal effect at 48 h (Figure 4B). SiO_2_TiO_2_ and Al_2_O_3_TiO_2_ NMs show a similar pattern of synergy, which was lower than the non-coated TiO_2_. Unexpectedly, AlSiTiO_2_ show a maximal synergistic effect only 72 h after irradiation.

We observed that the combination of irradiation with TiO_2_ NMS led to a synergistic effect indicating the enhancement of dose deposition and thus of electrons emission from the non-coated and coated TiO_2_ NMs.

### 2.6. TiO_2_ Induced Oxidative Stress

To further explain the increase in cell death observed after treatment with TiO_2_ NMs, and since TiO_2_ is known to have a photocatalytic effect, quantitative RT-PCR was performed on 16HBE14o- cells after TiO_2_ NMs treatment with or without 4 Gy irradiation using primers targeting genes involved in the response to oxidative stress. One of the most reliable and sensitive indicators of cellular oxidative stress is the 32 kDa heme-oxygenase-1 protein (HO-1). This protein, which is encoded by *HMOX1* gene, catabolizes the pro-oxidant heme into carbon monoxide (CO), free iron and biliverdin. Its expression, which is difficult to be detected in physiological conditions, can be induced upon oxidative stress or developed by reactive oxygen species (ROS) production and accumulation [25,26,27].

As a control of the basal level, RT-PCRs are also realized at T0, just after treatment and/or irradiation. At this time point, there was no difference in *HMOX1* expression in cells compared to the non-irradiated control group. However, as expected, this expression increased when cells were just treated with TiO_2_ NMs in a dose-dependent way from 24 h after introduction of NMs into cells until 72 h at least.

**Figure 5:**
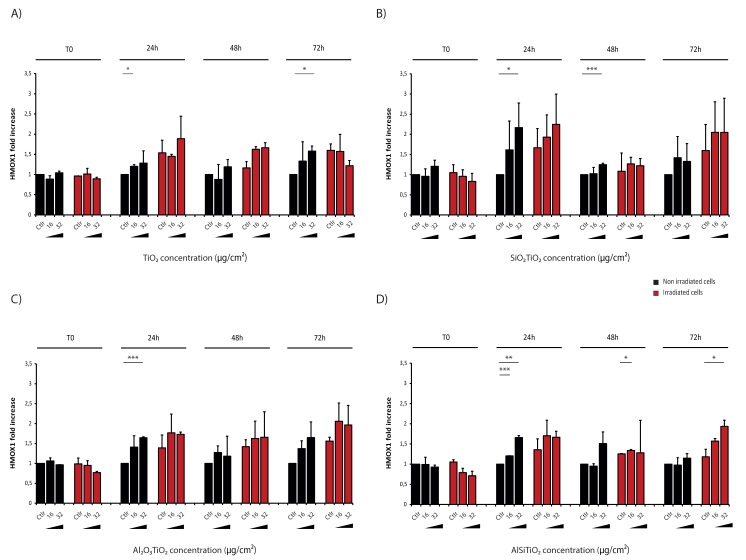
HMOX1 detection by quantitative real time polymerase chain reaction. Human bronchial epithelial 16HBE cells were maintained in control conditions (Ctlr) or treated with 16 or 32 g/cm of (**A**) titanium dioxide (TiO_2_), (**B**) silica coated titanium dioxide (SiO_2_TiO_2_), (**C**) aluminum coated titanium dioxide (Al_2_O_3_TiO_2_) or (**D**) double silica and aluminum coated titanium dioxide (AlSiTiO_2_), and 4 Gy irradiated or not. ARN extractions were performed immediately (T0), 24 h, 48 h or 72 h after treatment and quantitative RT-PCR were realized to detect HMOX1 gene expression. Representative dot plots normalized by the value of the untreated non-irradiated control for each time point are shown (means ± S.D., *n* = 2). * *p* < 0.05, ** *p* < 0.01, *** *p* < 0.001 (Student’s *t*-test), as compared with the control group corresponding.

Twenty-four hours after 4 Gy irradiation alone, an increase in *HMOX1* expression was measured, reflecting the induction of the oxidative stress due to the exposure. However, in the presence of non-coated TiO_2_ NMs under irradiation exposure, this expression was higher, meaning that cells undergo a higher stress, which could explain the higher level of cell death measured before (Figure 5A).

The same results were obtained with SiO_2_TiO_2_ and Al_2_O_3_TiO_2_ NMs, but it should be noticed that after 72 h of treatment, the coated NMs still induced *HMOX1* expression whereas non-coated TiO_2_ NMs did not, which illustrates, again, that the coated-TiO_2_ were more toxic than the non-coated TiO_2_ NMs, in these conditions (Figure 5A–C).

Surprisingly, in the case of AlSiTiO_2_, the increase of *HMOX1* expression induced by NMs after irradiation (compared to the IR control group) was only detectable from 72 h after treatment (Figure 5D).

Altogether, these results support the hypothesis that in the presence of TiO_2_ NMs, due to its photocatalytic properties, an irradiation exposure induces a higher level of oxidative stress in surviving cells, leading potentially to more damages into the cell and more activation of cell death pathways. 

Quantitative RT-PCR were also performed targeting two other genes: *NQO1*, coding for the NAD(P)H: quinone oxidoreductase 1, which is a two-electron reductase playing a role against oxidative damage via the reduction of endogenous quinone and *TXNRD1*, coding for the thioredoxin reductase, which is known to be playing an important role in the resistance to cytotoxic agents inducing oxidative stress [28,29]. The expression of these two genes is therefore part of the mechanisms of cell defense and then, supposed to be increased in case of oxidative stress induction.

Surprisingly, there is no induction of these gene expressions after treatment with the different types of TiO_2_ NMs_,_ suggesting that the ROS production induced by the TiO_2_ NMs is not the main cause of the observed cell death, at least 72 h after treatment (Figure S4).

## 3. Discussion

Currently in the field of nanomedicine, many studies are conducted to develop nanoparticles that could specifically sensitize radiation-resistant tumors [30]. All developments in this area are based on the photoactivation capacity of the NMs either made of high Z metal such as gold or having specific reactive surface like hydrogenated nanodiamonds [31,32]. Under radiation exposure, these nanomaterials have the capabilities of absorbing photons leading to electron emission that provokes radiation dose enhancement [31,32]. Overall, due to an increased interaction of photons with matter, NMs increased energy deposition within the cells and thus the ROS production is higher as compared to those generated by radiation alone. The challenge is to target NMs into tumors and minimize their entrance into normal cells to protect healthy tissues.

Here the question was different and was not yet addressed, we wondered if TiO_2_ NMs coming from non-controlled body contamination can be hazardous, through photocatalytic activity, for the healthy tissue of people receiving radiotherapy. In the case of radiotherapy, the highest dose is indeed delivered into the tumor, but healthy tissues in along the beam path are also exposed. Indeed, TiO_2_ NMs contaminations do occur since they are widely used in industrial and customer products, and people can be directly exposed by consuming foods and cosmetics, but also by inhalation [33]. The photocatalytic activity of TiO_2_ NMs has been well documented after non-ionizing radiation exposure (visible light and UVA) [34,35] but their photocatalytic activity after exposure to ionizing radiation such as γ- or X-rays used in radiotherapy and medical imaging are very little documented [36].

Since P25 TiO_2_ NMs is one of the most photochemical active TiO_2_ found in commercial products, we tested in the present work the cytotoxicity of various P25 TiO_2_ NMs non-coated, or coated with silica and/or alumina, the surface coating being utilized for a variety of applications, including enhanced protection from ultraviolet radiation while minimizing photocatalyst/oxidation activity by capturing radicals. The range of concentrations of TiO_2_ NMs used for cell exposure (6–64 µg/cm^2^) is compatible with those expected for a full working lifetime exposure to 20 nm spherical TiO_2_ NMs (49 µg/cm^2^) or for a 24 h exposure to 1 mg/cm^3^ aerosol in which TiO_2_ NMs size ranges from 5 to 100 nm [21].

We have shown that commercial TiO_2_ NMs alone non-coated and coated were already cytotoxic in a dose-dependent manner on the human normal bronchial epithelial 16HBE14o- cell line in the absence of any photo-activation since all incubation were done in the dark. Warheit et al. found that silica and alumina coated nanoparticles induce pulmonary toxicity, without any irradiation, due to their physical chemical properties [37]. The combined exposure of non-coated and coated TiO_2_ NMs with γ-rays increases significantly the radiation sensitivity of 16HBE14o- cells. We observed that the combination of irradiation with TiO_2_ NMS lead to a synergistic, implying that under irradiation, NMs retain a photocatalytic activity, which conjugated to the irradiation, accentuates the effects and lead to the activation of cell death pathways. These phenomena appear even faster when NMs are coated with silica, alumina or both probably because of their own cytotoxicity. The silica or alumina coated TiO_2_ NMs were synthetized for preventing photocatalyst activity while maintaining UV protection [20] during visible light and UVA exposure. In the case of ionizing radiation, photons have higher energy and consequently a weaker absorption of photons is expected. However, silica or alumina coating is not sufficient for preventing photocatalysis, knowing that a photo-electrochemical process was still shown under X-ray exposure [13]. The increase of HMOX1 gene expression, which is the leading indicator of oxidative stress, illustrates a higher induction of ROS production when cells are co-treated with NMs and IR. This can also explain the observed increased cell death. However, we should notify that the gene expressions implied in response to oxidative stress are not homogeneously induced after TiO_2_ NMs treatment, indicating that other pathways leading to cell death are activated, and should be clarified.

In a perspective of a better protection to non-ionizing radiation, industrial and consumers products use more and more P25 TiO_2_ coated with silicon or/and alumina. Even if these coatings provide a better protection in the case of non-ionizing radiation, they provide no protection in the case of a classical γ-rays radiation, but even increase the photocatalytic effect of the TiO_2_, and thus increase the damages on healthy cells.

The question of hazards should be addressed to a cohort of patients receiving irradiation. In this work, we have shown that at doses consistent with those used per day in a classical radiation therapy regimen, the presence of all types of TiO_2_ NMs radiosensitized human normal bronchial epithelial 16HBE14o- cells. The deleterious secondary effects of radiotherapy, which are associated with high toxicity on healthy tissues, can be explained in rare cases with patient’s genetic susceptibilities but most often remain unexplained. Contaminations by NMs presenting a photocatalytic activity, such as TiO_2_ NMs, which is widespread in usual products, may be at the origin of some of these cases of high radiation sensitivity. The hazards of TiO_2_ NMs contamination regarding the sensitivity of healthy tissues and tumors in response to low doses, used for diagnostic imaging, and high doses of ionizing radiation deserve to be explored.

## 4. Material and Methods

### 4.1. Materials

TiO_2_ P25 NMs were purchased from Sigma-Aldrich^®^ (Aeroxide^®^ P25). The primary given diameter is 25 nm of material composed of 85% of the anatase form and 15% of the rutile form (more than 99.5% purity according to manufacturer information). NMs were suspended in ultrapure water and dispersed according to the EU Framework 7 Program Nanogentotox recommendations, at 10 mg/mL by sonication (24 kHz, 240 W, Hielscher UP400S, sonotrodeH3, cycle 0.5, power 30%) for 1 h under a cooling system.

Suspensions were characterized by Dynamic Light Scattering (DLS) on a Nanosizer ZS (Malvern) in the back-scattering configuration (173°). Sizes and zeta potentials were 169 nm and +32.7 mV, 380 nm and −10 mV and 419 nm and −10 mV in water, complete complete DMEM (Dulbecco’s Modified Eagle Medium).

It should be noticed that the hydrodynamic diameter of the particle is higher in both water and culture medias as compared to the one given by the manufacturer. This is due to aggregation (in water and media) and protein corona formation (in media) that usually occurs following the suspension step of nanoparticles.

Titanium silicon oxide (TiSiO_4_) NMs and aluminum titanate (Al_2_O_3_.TiO_2_) NMs were purchased from Sigma-Aldrich^®^ (St. Louis, MO, USA), at the particle size under 50 nm and 25 nm, respectively. Rutile titanium dioxide coated with silicon and aluminum (AlSiTiO_2_) NMs were purchased from US Research Nanomaterials (Houston, TX, USA), at the primary diameter of 30 nm. All the coated TiO_2_ were suspended as described above.

### 4.2. Cell Line and Cell Culture

Unless indicated, media, antibiotics and supplements were purchased from Gibco-Life Technologies (Carlsbad, CA, USA), and chemicals from Sigma-Aldrich (St Louis, MO, USA). The human bronchial epithelial 16HBE14o- cell line was cultured in modified Eagle medium (MEM) supplemented with 10% heat-inactivated fetal bovine serum, 1% l-Glutamine, 10 U/mL penicillin sodium and 10 µg/mL streptomycin sulfate. The flasks used for 16HBE14o- were coated for 3 h at 37 °C with 2 mL of LHC basal medium supplemented with 0.01% bovine serum albumin purchased from Sigma, 0.003% Vitrogen 100 purchased from BD, 0.001% human fibronectin purchased from Sigma-Aldrich. Cells were cultured at 37 °C in a humidified atmosphere of 5% CO_2_ and 95% air and in dark to avoid any light and UV photocatalysis activation.

### 4.3. Cell Exposure

According to preliminary dose-ranging studies, after 24 h of culture for 16HBE14o- in 96 well microplates, cells were exposed to 6, 16, 32, 48 and 64 µg/cm^2^ (10, 25, 50, 75 and 100 µg/mL) of the different TiO_2_ NMs, for 0, 24, 48 and 72 h [38]. Surface area was used in all our experiments seeing that it appears to be the most appropriate metric to assess NM toxicity *in vivo* and *in vitro* studies to keep constant the ratio amount of NMs per surface unit of cell culture [39]. 

### 4.4. γ-Ionizing Irradiation

Cells were irradiated immediately after being exposed to TiO_2_ NMs in a 137Cs source irradiation unit (IBM637, CisBio International, Gif sur Yvette, France) at 1.61 Gy·min^−1^. The dose of 4 Gy was chosen as a low-toxic dose according to a dose response curve previously defined.

### 4.5. Flow Cytometry

Cell death was monitored by the uptake of the dyes DiOC_6_(3) (3,3′-Dihexyloxacarbocyanine Iodide; Thermo Fisher, Waltham, MA, USA) and propidium iodide (PI; Sigma Aldrich, Saint-Louis, MI, USA). To this aim, immediately (T0), 24, 48 and 72 h after exposure to TiO_2_ NMs combined or not with 4 Gy irradiation, cells were incubated in culture medium supplemented with 40 nM DiOC_6_(3) and 1 µg/mL PI for 30 min at 37 °C. Cytofluorometric acquisitions were performed on a BD Facscalibur (BD Biosciences, San Jose, CA, USA). Data were analyzed using FlowJo^®^ software (https://www.flowjo.com).

### 4.6. Fluorescence Microscopy

16HBE14o- cells were platted at 25 × 103 cells per well on 24-well plates and after 24 h, they were treated with 0, 6, 16, 32 or 64 µg/cm^2^ of the different types of TiO_2_ NMs. Microscopic images were acquired from fixed cells using Axiovert 200 fluorescent microscope (Zeiss, Oberkochen, Germany), immediately (D0), 1 day (D1), 2 days (D2), 3 days (D3), 6 days (D6) and 7 days (D7) after treatment with TiO_2_ NMs with or without 4 Gy irradiation. Cells were fixed 10 min in a PBS solution supplemented with 4% of paraformaldehyde (PFA) and 10% of Hoechst 33,342 (Molecular Probes_Life Technologies), then washed 3 times with PBS, and kept at 4 °C until used. Cell counting was automatically performed using the EBImage package (https://www.bioconductor.org/) implemented for R language (https://cran.r-project.org/).

### 4.7. Quantitative Reverse Transcription Polymerase Chain Reaction

16HBE14o- cells were treated or not with the NMs at 16 or 32 µg/cm^2^, combined or not with 4 Gy irradiation. Cell pellets were collected immediately (T0), 24, 48 or 72 h after treatment. RNA extractions were realized with RNA InstaPure^TM^ System (Eurogentec, Liège, Belgique). After homogenization of the cell pellets with 1 mL of RNA Instapure, RNA was extracted by the addition of a 0.2 volume of chloroform, then precipitated with 1 volume of isopropanol, and finally washed 2 times with 70% ethanol as per manufacturer’s instructions. RNA quantity was measured by a NanoDrop (Thermo Fisher) and RNA quality was confirmed by agarose gel migration. cDNA synthesis was realized using the SuperScript^TM^ VILO^TM^ cDNA Synthesis Kit (Invitrogen, Carlsbad, CA, USA) as instructed per the manufacturer’s instructions. PCR probes for HMOX detection (Hs01110250_m1), NQO1 detection (Hs02512143_s1) and TXNRD1 detection (Hs00917067) were provided by Applied Biosystems (Foster city, CA, USA). RT-PCR was realized on a 7300 Real Time PCR System (Applied Biosystems, Foster city, CA, USA).

### 4.8. Statistical Analysis

A Welch student’s *t*-test was performed for cell death experiments, each irradiated condition compared to non-irradiated cells, and each dose of NMs applied on irradiated cells compared to irradiated control cells.

### 4.9. Statistical Analysis Qualifiying the NMs Effect on Radiation Response

In order to investigate if there is a synergistic effect between NMs and irradiation, a mathematical model simulating the theoretical cell death of 4 Gy irradiation added to the different types of TiO_2_ NM was performed. The model was based on the results of cell death obtained with the different conditions separately “NMs alone” and “4 Gy IR alone”. For each condition, a dose-response model was built using a 4-parameter log-logistic (LL4) fit [24] by means of drc R package. The 4-parameter logistic model generalizes the usual logistic regression model to allow the lower and upper response asymptotes to be greater than zero and less than one, respectively, the four parameters being (a) the baseline response probability (at dose 0), (b) the maximum response probability (at an infinite dose), (c) the dose producing a response probability halfway between (a) and (b) and (c) the shape parameter.

For each type of NM, an irradiated (IR) dose-response model was then simulated from the non-irradiated (NI) response by transferring the IR impulsional response to each NI condition. Synergistic killing effect of irradiation together with NMs treatment was assessed by comparing the LL4 model coefficients from the dose-response models of the IR-simulated response versus IR biological response. This comparison was performed by calculating the Euclidian distance between the cited coefficients.

## Supplementary Materials

The following are available online at https://www.mdpi.com/1422-0067/21/3/940/s1.

## References

[1] Baan R., Straif K., Grosse Y., Secretan B., El Ghissassi F., Cogliano V., WHO International Agency for Research on Cancer Monograph Working Group (2006). Carcinogenicity of carbon black, titanium dioxide, and talc. Lancet. Oncol..

[2] Luo Z., Wang Z., Xu B., Sarakiotis I.L., Du Laing G., Yan C. (2014). Measurement and characterization of engineered titanium dioxide nanoparticles in the environment. Appl. Phys. Eng..

[3] Piccinno F., Gottschalk F., Seeger S., Nowack B. (2012). Industrial production quantities and uses of ten engineered nanomaterials in Europe and the world. J. Nanoparticle. Res..

[4] Auffan M., Rose J., Wiesner M.R., Bottero J.Y. (2009). Chemical stability of metallic nanoparticles: A parameter controlling their potential cellular toxicity in vitro. Env. Pollut..

[5] Ponce A. (2013). Nanomaterials and workplace health & safety What are the issues for workers?. http://www.etui.org/Publications2/Guides/Nanomaterials-and-workplace-health-safety.-What-are-the-issues-for-workers.

[6] Shi H., Magaye R., Castranova V., Zhao J. (2013). Titanium dioxide nanoparticles: A review of current toxicological data. Part. Fibre. Toxicol..

[7] Long T.C., Saleh N., Tilton R.D., Lowry G.V., Veronesi B. (2006). Titanium dioxide (P25) produces reactive oxygen species in immortalized brain microglia (BV2): Implications for nanoparticle neurotoxicity. Env. Sci. Technol..

[8] Jovanovic B. (2015). Review of titanium dioxide nanoparticle phototoxicity: Developing a phototoxicity ratio to correct the endpoint values of toxicity tests. Env. Toxicol. Chem..

[9] Li Y., Zhang W., Niu J., Chen Y. (2012). Mechanism of photogenerated reactive oxygen species and correlation with the antibacterial properties of engineered metal-oxide nanoparticles. ACS Nano..

[10] Sanders K., Degn L.L., Mundy W.R., Zucker R.M., Dreher K., Zhao B., Roberts J.E., Boyes W.K. (2012). In vitro phototoxicity and hazard identification of nano-scale titanium dioxide. Toxicol. Appl. Pharm..

[11] Xue C., Wu J., Lan F., Liu W., Yang X., Zeng F., Xu H. (2010). Nano titanium dioxide induces the generation of ROS and potential damage in HaCaT cells under UVA irradiation. J. Nanosci. Nanotechnol..

[12] Ackroyd R., Kelty C., Brown N., Reed M. (2001). The history of photodetection and photodynamic therapy. Photochem. Photobiol..

[13] Tamura K., Ohko Y., Kawamura H., Yoshikawa H., Tatsuma T., Fujishima A., Mizuki J. (2007). X-ray induced photoelectrochemistry on TiO2. Electrochim. Acta.

[14] Mirjolet C., Papa A.L., Crehange G., Raguin O., Seignez C., Paul C., Truc G., Maingon P., Millot N. (2013). The radiosensitization effect of titanate nanotubes as a new tool in radiation therapy for glioblastoma: A proof-of-concept. Radiother. Oncol..

[15] Townley H.E., Kim J., Dobson P.J. (2012). In vivo demonstration of enhanced radiotherapy using rare earth doped titania nanoparticles. Nanoscale.

[16] Chen T., Yan J., Li Y. (2014). Genotoxicity of titanium dioxide nanoparticles. J. Food Drug Anal..

[17] Iavicoli I., Leso V., Fontana L., Bergamaschi A. (2011). Toxicological effects of titanium dioxide nanoparticles: A review of in vitro mammalian studies. Eur. Rev. Med. Pharm. Sci..

[18] Dankovic D., Kuempel E., Wheeler M. (2007). An approach to risk assessment for TiO2. Inhal. Toxicol..

[19] Borm P.J., Albrecht C. (2004). Inhaled particles and lung cancer. Part A: Mechanisms. Int. J. Cancer.

[20] Egerton T. (2013). The Influence of Surface Alumina and Silica on the Photocatalytic Degradation of Organic Pollutants. Catalysts.

[21] Gangwal S., Brown J.S., Wang A., Houck K.A., Dix D.J., Kavlock R.J., Hubal E.A. (2011). Informing selection of nanomaterial concentrations for ToxCast in vitro testing based on occupational exposure potential. Env. Health Perspect..

[22] Zucker R.M., Massaro E.J., Sanders K.M., Degn L.L., Boyes W.K. (2010). Detection of TiO2 nanoparticles in cells by flow cytometry. Cytom. A..

[23] Kepp O., Galluzzi L., Lipinski M., Yuan J., Kroemer G. (2011). Cell death assays for drug discovery. Nat. Rev. Drug Discov..

[24] Dinse G. (2011). An EM Algorithm for Fitting a 4-Parameter Logistic Model to Binary Dose-Response Data. J. Agric. Biol. Env. Stat..

[25] Gozzelino R., Jeney V., Soares M.P. (2010). Mechanisms of cell protection by heme oxygenase-1. Annu Rev. Pharm. Toxicol..

[26] Poss K.D., Tonegawa S. (1997). Reduced stress defense in heme oxygenase 1-deficient cells. Proc. Natl. Acad. Sci. USA.

[27] Salerno L., Romeo G., Modica M.N., Amata E., Sorrenti V., Barbagallo I., Pittala V. (2017). Heme oxygenase-1: A new druggable target in the management of chronic and acute myeloid leukemia. Eur. J. Med. Chem..

[28] Nguyen P., Awwad R.T., Smart D.D., Spitz D.R., Gius D. (2006). Thioredoxin reductase as a novel molecular target for cancer therapy. Cancer Lett..

[29] Ross D., Kepa J.K., Winski S.L., Beall H.D., Anwar A., Siegel D. (2000). NAD(P)H:quinone oxidoreductase 1 (NQO1): Chemoprotection, bioactivation, gene regulation and genetic polymorphisms. Chem. Biol. Interact..

[30] Wang H., Mu X., He H., Zhang X.-D. (2018). Cancer Radiosensitizers. Trends Pharmacol. Sci..

[31] Kumar R., Korideck H., Ngwa W., Berbeco R.I., Makrigiorgos G.M., Sridhar S. (2013). Third generation gold nanoplatform optimized for radiation therapy. Transl. Cancer Res..

[32] Ngwa W., Kumar R., Sridhar S., Korideck H., Zygmanski P., Cormack R.A., Berbeco R., Makrigiorgos G.M. (2014). Targeted radiotherapy with gold nanoparticles: Current status and future perspectives. Nanomedicine (London).

[33] Baranowska-Wójcik E., Szwajgier D., Oleszczuk P., Anna W.-M. (2020). Effects of Titanium Dioxide Nanoparticles Exposure on Human Health-a Review. Biol. Trace. Elem. Res..

[34] Lee K., Yoon H., Ahn C., Park J., Jeon S. (2019). Strategies to improve the photocatalytic activity of TiO2: 3D nanostructuring and heterostructuring with graphitic carbon nanomaterials. Nanoscale.

[35] Noman M.T., Ashraf M.A., Ali A. (2019). Synthesis and applications of nano-TiO2: A review. Environ. Sci. Pollut. Res. Int..

[36] Bello L.M.P., Williams P., Reece P., Lumpkin G.R., Sheppard L.R. (2014). Study of gamma irradiation effect on commercial TiO2 photocatalyst. Appl. Radiat. Isot..

[37] Warheit D., Brock W.J., Lee K.P., Webb T.R., Reed K.L. (2005). Comparative pulmonary toxicity inhalation and instillation studies with different TiO2 particle formulations: Impact of surface treatments on particle toxicity. Toxicol. Sci..

[38] Cho W.S., Duffin R., Poland C.A., Howie S.E., MacNee W., Bradley M., Megson I.L., Donaldson K. (2010). Metal oxide nanoparticles induce unique inflammatory footprints in the lung: Important implications for nanoparticle testing. Environ. Health. Perspect..

[39] Duffin R., Tran L., Brown D., Stone V., Donaldson K. (2007). Proinflammogenic effects of low-toxicity and metal nanoparticles in vivo and in vitro: Highlighting the role of particle surface area and surface reactivity. Inhal. Toxicol..

