# Supplementary material for: TiO2 Nanomaterials Non-Controlled Contamination Could Be Hazardous for Normal Cells Located in the Field of Radiotherapy
Source: Int J Mol Sci. 2020 Jan 31;21(3):940. doi: 10.3390/ijms21030940 (PMC7037422; doi:10.3390/ijms21030940)
Supplement: Supplementary file 1 [file ijms-21-00940-s001.zip › Fig S1a internalization 48h.pdf]

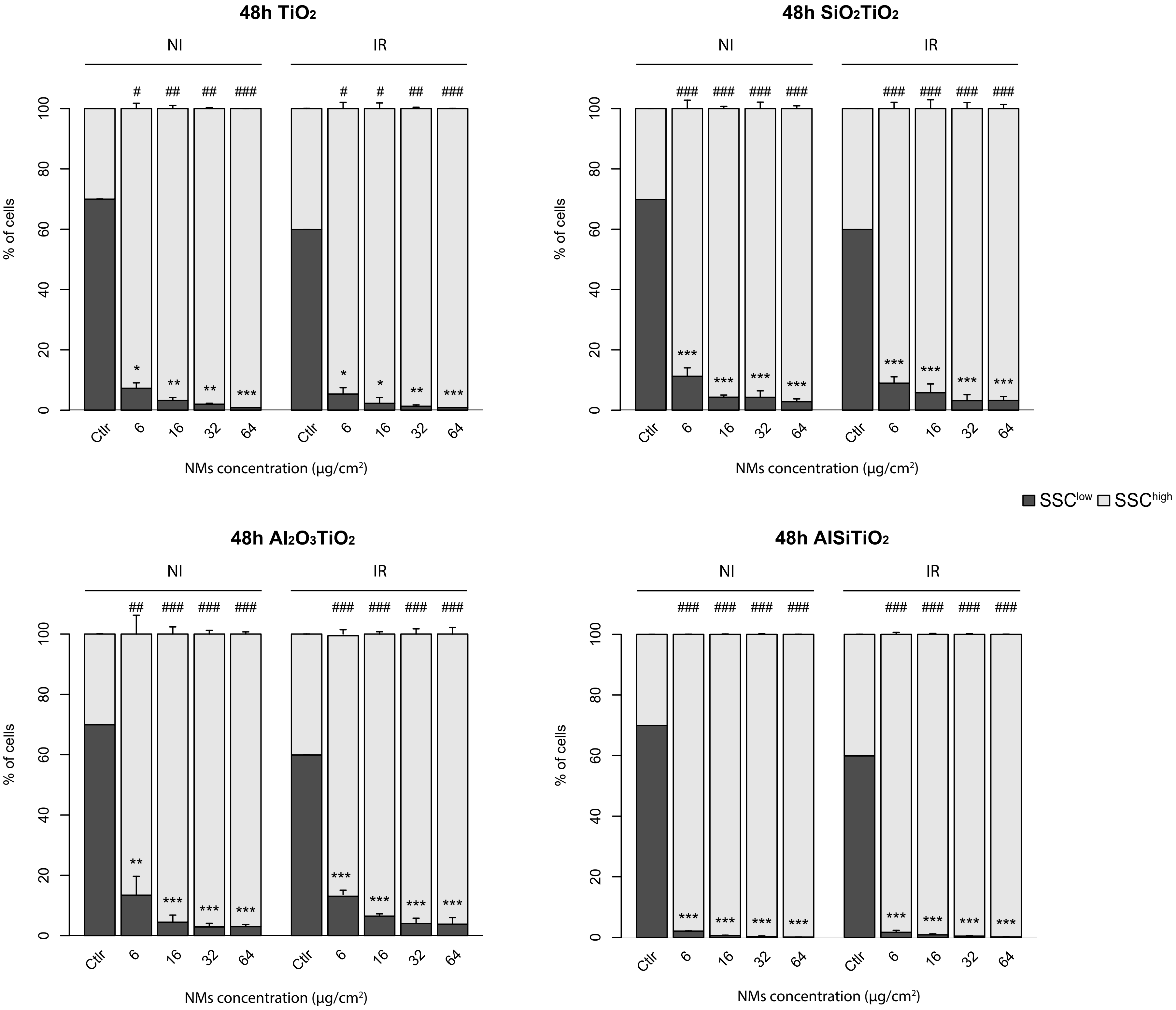

Fig. S1a: NMs internalization into 16HBE cells at 48h after treatment.

Same tresholds are applied and the percentage of SSC^{low} and SSC^{high} cells are represented here. (means ± S.E.M., n=3, $^{***}P<0.001$ (Welch t-test), as compared to the percentage of SSC^{low} of the control group, $^{\#\#\#}P<0.001$ (Welch t-test), as compared to the percentage of SSC^{high} of the control group.)
