# Supplementary material for: TiO2 Nanomaterials Non-Controlled Contamination Could Be Hazardous for Normal Cells Located in the Field of Radiotherapy
Source: Int J Mol Sci. 2020 Jan 31;21(3):940. doi: 10.3390/ijms21030940 (PMC7037422; doi:10.3390/ijms21030940)
Supplement: Supplementary file 1 [file ijms-21-00940-s001.zip › Fig S2 TiO2 DiOC IP.pdf]

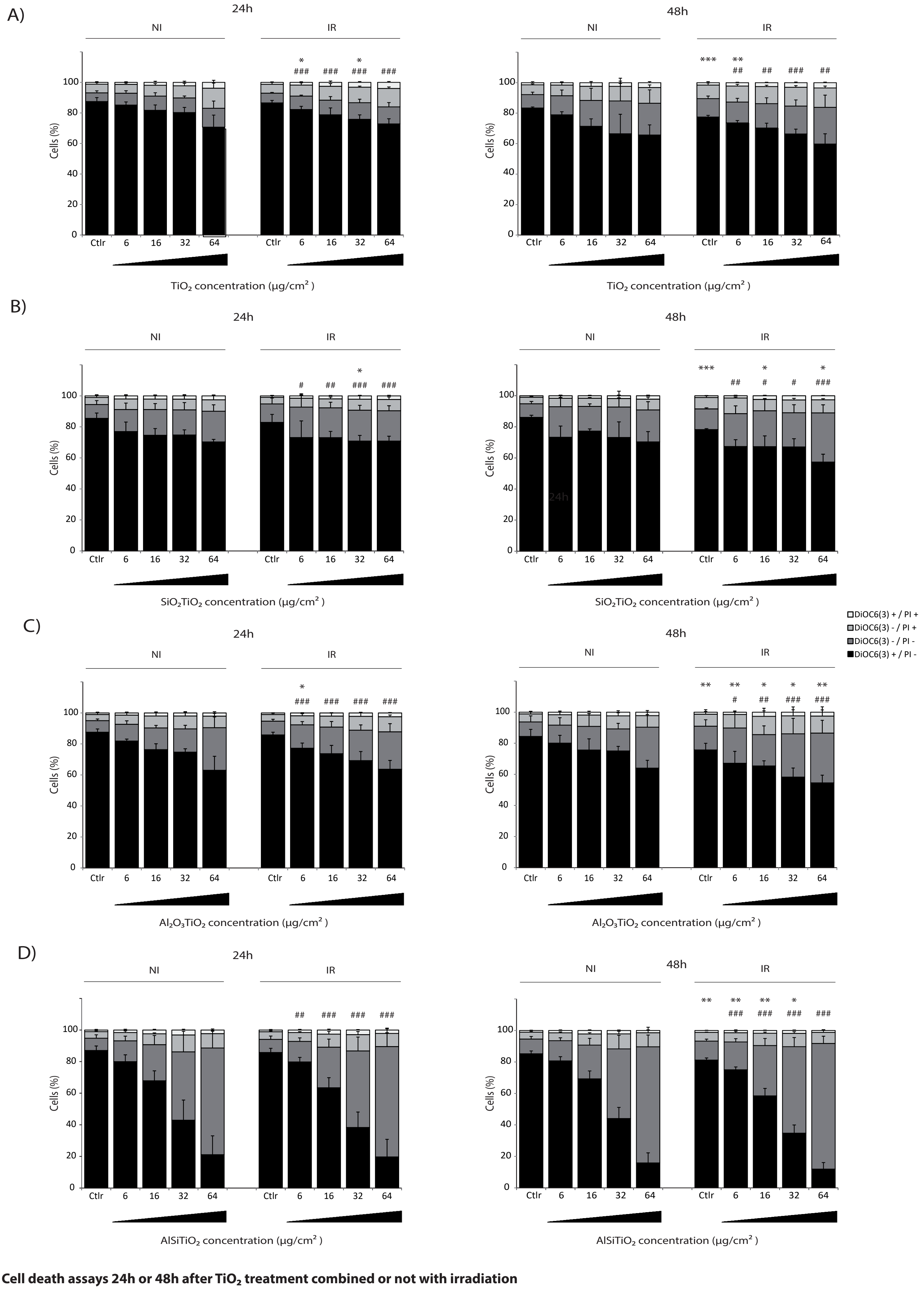

Fig S2. Cell death assays 24h or 48h after TiO_2 treatment combined or not with irradiation

Representative dot plots and quantitative data are reported (means ± S.D, n=3, except for A) 24h n=4, A) 48h and B) 24h n=4). *P<0.05, **P<0.01, ***P<0.001 (Student's t-test), as compared with cells maintained in same conditions but non-irradiated. #P<0.05, ##P<0.01, ###P<0.001 (Student's t-test), as compared with IR cells maintained in control (Ctlr) condition.)
