# Supplementary material for: TiO2 Nanomaterials Non-Controlled Contamination Could Be Hazardous for Normal Cells Located in the Field of Radiotherapy
Source: Int J Mol Sci. 2020 Jan 31;21(3):940. doi: 10.3390/ijms21030940 (PMC7037422; doi:10.3390/ijms21030940)
Supplement: Supplementary file 1 [file ijms-21-00940-s001.zip › Fig S4 Results Synergy.pdf]

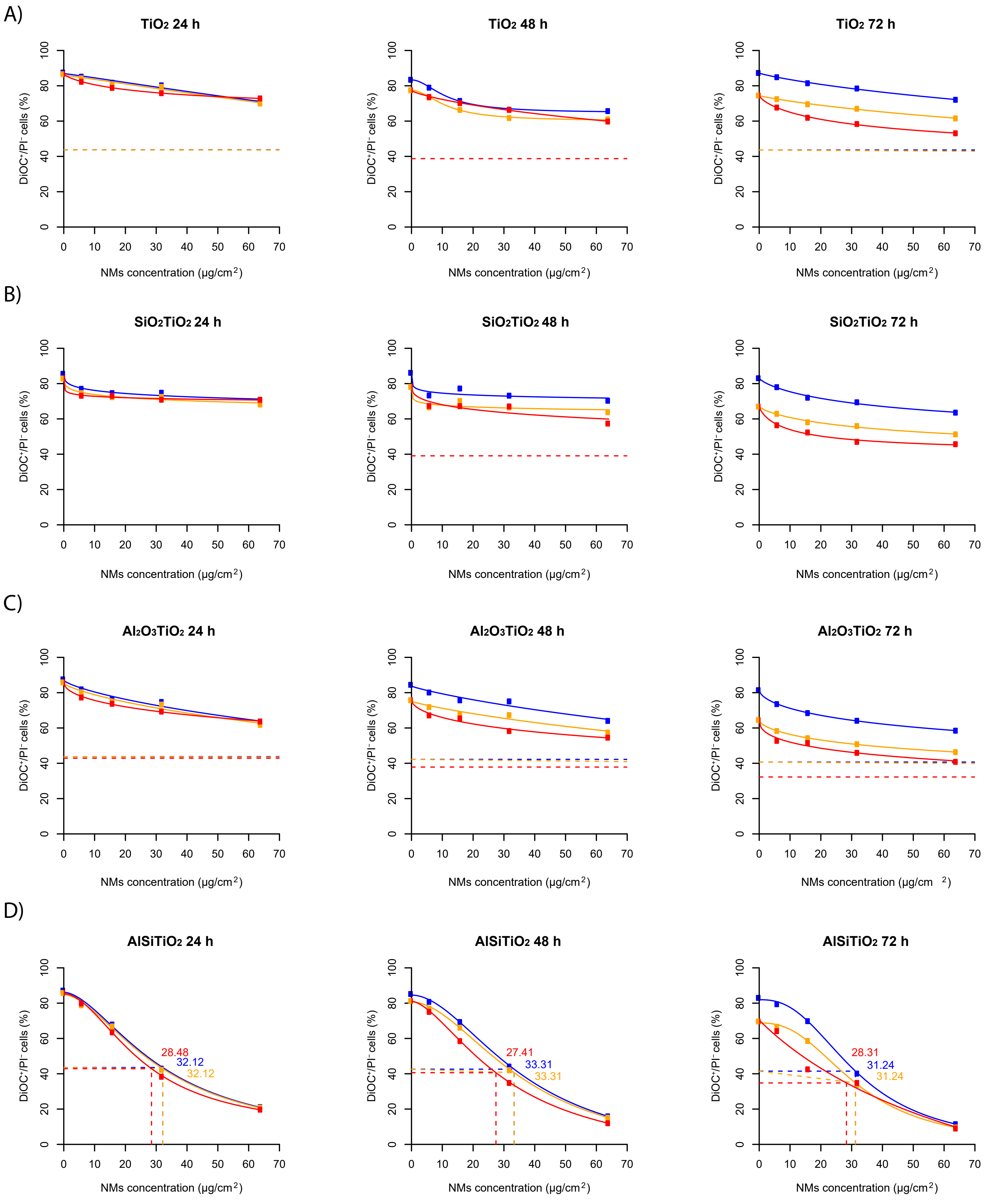

Fig. S4: Synergy study between each type of TiO_2 and a 4Gy irradiation.

The log-logistic model simulates the theoretical effect between a 4Gy irradiation and A) TiO_2, B) SiO_2TiO_2, C) $Al_2O_3TiO_2$, D) $AlSiTiO_2$, and fits the real effect obtained experimentally for 24h, 48h and 72h after treatment.
