# Supplementary material for: TiO2 Nanomaterials Non-Controlled Contamination Could Be Hazardous for Normal Cells Located in the Field of Radiotherapy
Source: Int J Mol Sci. 2020 Jan 31;21(3):940. doi: 10.3390/ijms21030940 (PMC7037422; doi:10.3390/ijms21030940)
Supplement: Supplementary file 1 [file ijms-21-00940-s001.zip › Fig S5 TiO2 PCR.pdf]

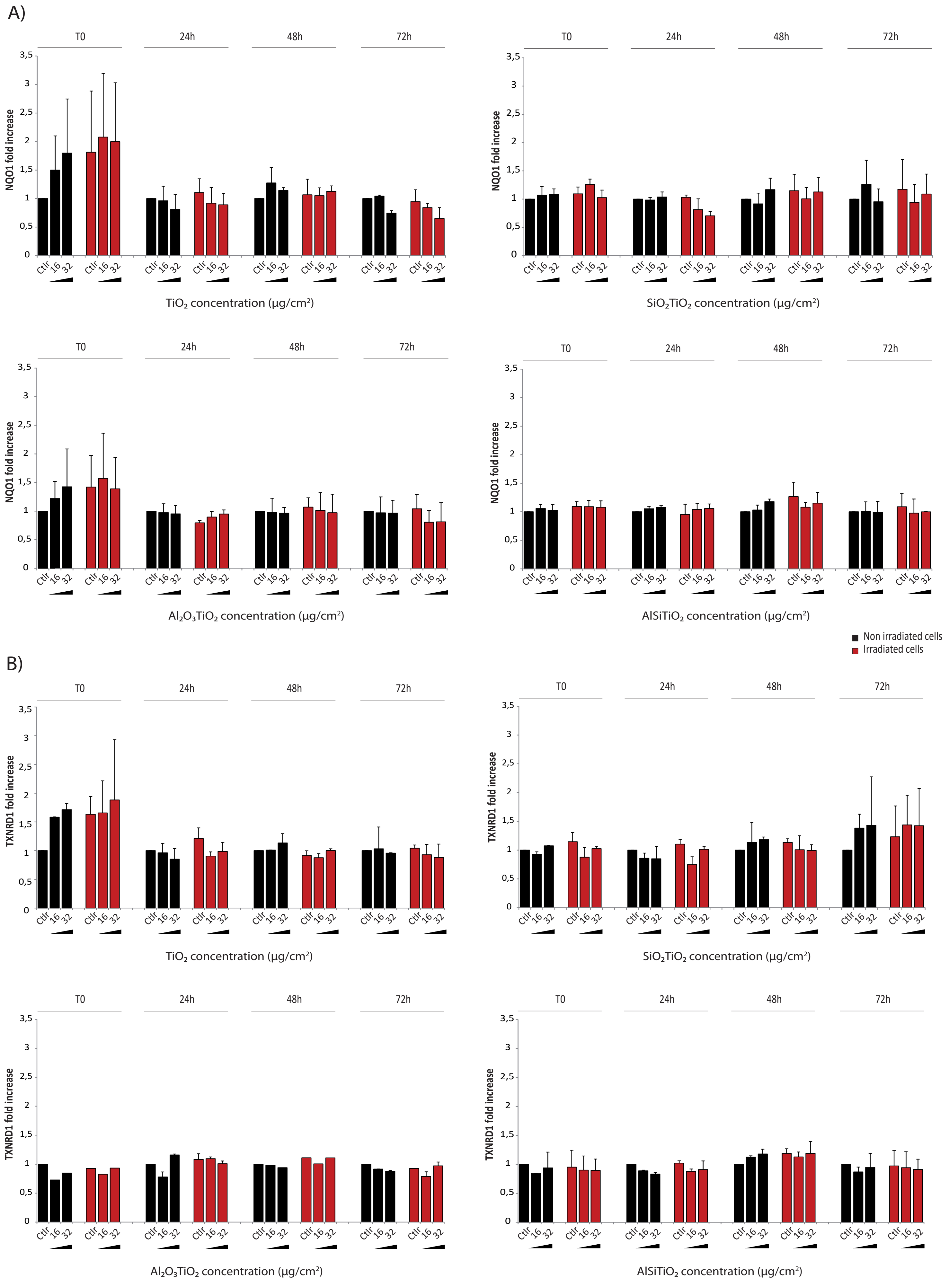

Fig. S5: Detection of NQO1 and TXNRD1 expression by quantitative RT-PCR.

A) Detection of NQO1 gene expression and B) TXNRD1 gene expression immediately (T0), 24h, 48h or 72h after treatment with or without a 4Gy irradiation.

Representative dot plots normalized by the value of the untreated non-irradiated control for each time point (means ± S.D., n=2).
